# Structural Characterization of Disaccharides Using Cyclic Ion Mobility Spectrometry and Monosaccharide Standards

**DOI:** 10.1021/jasms.4c00029

**Published:** 2024-04-18

**Authors:** Bram van de Put, Wouter J.C. de Bruijn, Henk A. Schols

**Affiliations:** Laboratory of Food Chemistry, Wageningen University, Bornse Weilanden 9, 6708, WG Wageningen, The Netherlands

## Abstract

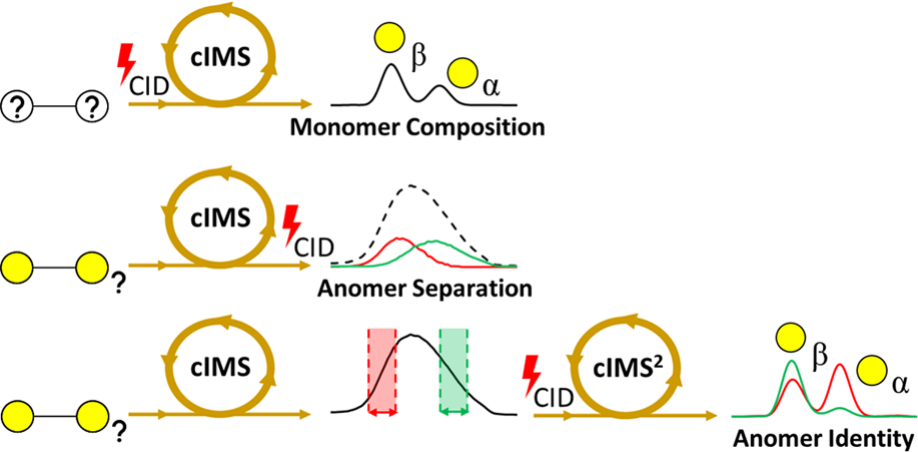

To understand the mode of action of bioactive oligosaccharides, such as prebiotics, in-depth knowledge about all structural features, including monosaccharide composition, linkage type, and anomeric configuration, is necessary. Current analytical techniques provide limited information about structural features within complex mixtures unless preceded by extensive purification. In this study, we propose an approach employing cyclic ion mobility spectrometry (cIMS) for the in-depth characterization of oligosaccharides, here demonstrated for disaccharides. We were able to separate galactose and glucose anomers by exploiting the high ion mobility resolution of cIMS. Using the obtained monosaccharide mobilograms as references, we determined the composition and anomeric configuration of 4β-galactobiose by studying the monosaccharide fragments generated by collision-induced dissociation (CID) before the ion mobility separation. Drift times and individual MS^2^ spectra of partially resolved reducing-end anomers of 4β-galactobiose, 4β-galactosylglucose (lactose), and 4β-glucosylglucose (cellobiose) were obtained by deconvolution using CID fragmentation induced in the transfer region between the cIMS cell and TOF analyzer. The composition and anomeric configuration of the reducing end anomers of these disaccharides were identified using cIMS^2^ approaches, where first each anomer was isolated using cIMS and individually fragmented, and the monosaccharide fragments were again separated by cIMS for comparison with monosaccharide standards. With these results we demonstrate the promising application of cIMS for the structural characterization of isomeric oligosaccharides.

## Introduction

The structures of oligosaccharides are difficult to analyze due to their high variability. Every oligosaccharide can consist of different monosaccharides, each present in either the α- or β-anomeric configuration, and can be connected by a variety of linkage types.^1^ These structural variations result in only small differences in the physicochemical properties, making oligosaccharide isomers hard to separate and identify. Combined with the fact that many oligosaccharide formulations consist of tens or even hundreds of unique oligosaccharide structures, the characterization of all individual oligosaccharide isomers is challenging.

Structural characterization is particularly relevant for bioactive oligosaccharides, such as prebiotic lactose-based galactooligosaccharides (GOSs). GOS is one of the most studied prebiotic oligosaccharides and is credited with a number of positive health effects including modulating the immune system,^2,3^ steering the composition of the gut microbiota,^4−6^ altering the metabolic activity of the gut microbiota,^7−9^ and supporting the development of the immune system of neonates.^10−13^ Due to the diversity of the oligosaccharide structures present in prebiotic and immune-stimulating supplements, the precise structural features of the individual oligosaccharides responsible for specific health benefits remain mostly unknown.^14^

The most in-depth characterization studies first separate complex oligosaccharide mixtures by size-exclusion chromatography to obtain fractions of a single degree of polymerization, followed by an interaction-based preparative chromatographic separation to obtain individual isomers that can then be characterized by NMR spectroscopy.^15−17^ However, preparative chromatography is not capable of separating all of the isomers effectively. Furthermore, it is a time-consuming and costly process to obtain sufficient purified material for NMR, and thus typically only the most abundant structures can be studied.

LC-MS/MS approaches are promising for both fast and in-depth analysis of oligosaccharide mixtures,^18−20^ as LC provides fast separation of many isomeric forms and MS/MS fragmentation patterns provide structural information. Generally, a set of known reference compounds representing the structural features of interest is tested to establish fragmentation rules. For example, Hernandez-Hernandez et al. studied GOS with HILIC-MS^n^ using disaccharide references with known composition and linkage type to establish characteristic neutral loss ratios.^21^ They studied three commercial GOS formulations and were able to separate up to 17 structures per sample. Some linkage types were proposed for the separated isomers by comparing their neutral loss ratios to those of the disaccharide standards. However, due to the unpredictable nature of MS fragmentation ratios, the assignments are suggestive. Van Leeuwen et al. fractionated a GOS sample with size exclusion chromatography followed by anion exchange chromatography and isolated 44 structures, which they characterized using NMR.^22^ Nonetheless, as shown by Logtenberg et al., who studied GOS using PGC-MS^2^ and recognized 107 individual structures,^20^ there are still many structures to be fully identified. Logtenberg et al.^20^ further demonstrated that fragmentation rules established with standards can be used to identify 1,3-, 1,4-, and 1,6-linkage types for the nonreducing end of the sugar moiety of unknown trisaccharides. However, due to the reduction step necessary to prevent anomerization for effective chromatographic separation, information on the reducing end linkage type was lost. Furthermore, Logtenberg et al. could not distinguish monosaccharide identities and assumed the presence of glucose at the reducing end and galactose at all other positions within a reducing trisaccharide.^20^

Despite these developments in the analysis of GOS, no fragmentation rules have been found to confidently identify monomer types and anomeric configurations. As a result, analyzing the structural composition of complex oligosaccharide mixtures with established methodologies remains challenging. At the same time, ion mobility spectrometry (IMS) has gained a lot of interest in recent years, in particular for its ability to identify structural motifs by relating fragment mobilograms to those of known standards. As an example, Bansal et al. demonstrated the use of IMS^2^ to record cryogenic IR spectra of characteristic fragments of human milk oligosaccharide structures.^23^ Additionally, Ollivier et al. used cyclic IMS (cIMS) to identify the anomeric configuration of the mannose linkages within tri- and tetra-manno-oligosaccharides by comparison with disaccharide fragments of ^18^O-labeled manno di- and trisaccharide standards of known anomeric configuration.^24^ These studies have demonstrated several prerequisites for the use of ion mobility spectrometry for the unambiguous characterization of unknown oligosaccharides: (1) relevant oligosaccharide motifs are sufficiently separable,;^25^ (2) the mobilities of oligosaccharide fragments are identical to those of standards with the same structure,^26^ (3) the anomeric configuration does not change upon fragmentation, and (4) standards that represent the structural features under investigation are necessary, although these will always exist as an equilibrium mixture of reducing-end anomers. Recently, Ollivier et al. demonstrated that homo-oligosaccharide compositions can be probed using cIMS by comparing the mobilograms of their monosaccharide fragments with monosaccharide standards.^27^ This forms a strong basis for the *de novo* sequencing of oligosaccharides. However, when using monosaccharides as standards, it is only possible to determine composition and anomeric configuration but not linkage type.

In this research, we separated and identified disaccharide anomers using high-resolution cIMS employing underivatized monosaccharides as standards as a proof of concept for complete *de novo* sequencing of oligosaccharides. To this end, we investigated the application of pre-cIMS CID for fast compositional identification of disaccharides. Additionally, we developed a method to determine drift times of disaccharide reducing-end anomers by deconvolution using post-cIMS CID. The anomeric configuration of the overlapping reducing end anomers was determined by cIMS^2^. We applied our newly developed approaches to fully identify the compositions and anomeric configurations of 4β-galactobiose, lactose, and cellobiose, which are important building blocks of prebiotic galactooligosaccharides.

## Experimental Section

### Chemicals and Reagents

**Table 1:**
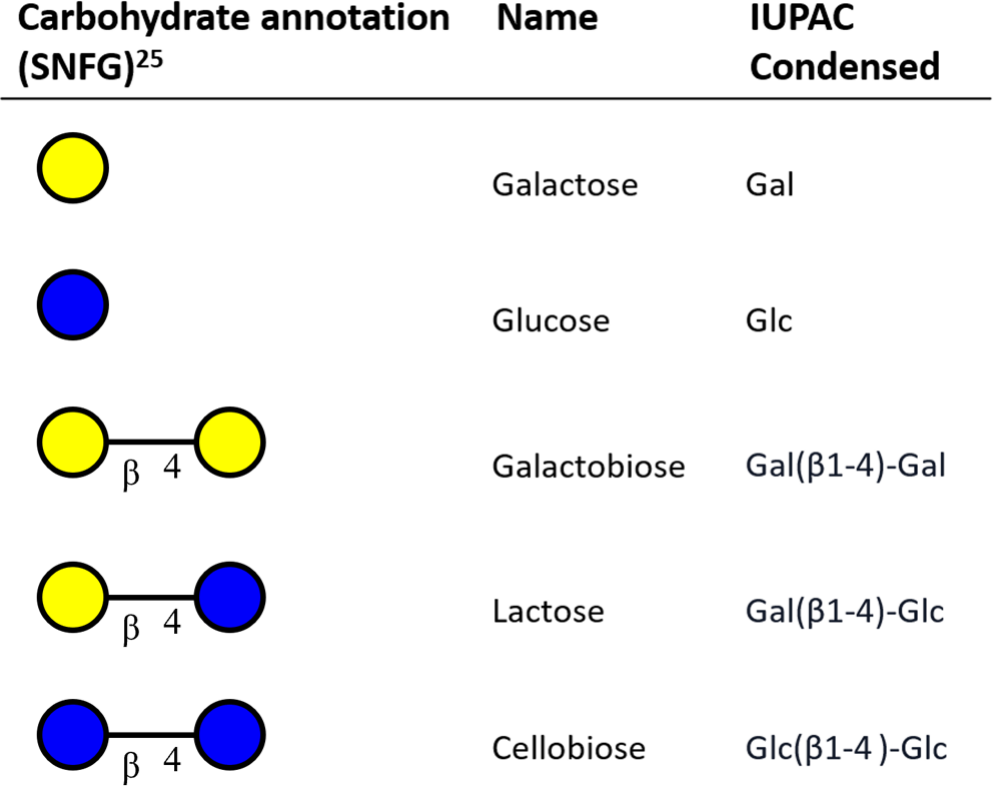
Structures and Names of GOS-Analogous Mono- and Disaccharides Used in This Study

### Cyclic Ion Mobility Spectrometry

A SELECT SERIES Cyclic IMS system (Waters Co., Wilmslow, UK) was used for all analyses. The internal fluidic system of the cIMS system was used for direct infusion experiments at a flow rate of 3 μL/min. ESI source settings were as follows: capillary potential of 2.5 kV, cone potential of 10 V, nebulizer pressure of 6 bar, cone gas of 50 L/h, desolvation gas flow of 200 L/h, desolvation temperature of 350 °C, and source temperature of 150 °C. Negative mode ionization was found to be inefficient at the low flow rates achievable with the built-in direct infusion fluidics of the cIMS system. The negative polarity experiments were therefore conducted using flow injection on an ACQUITY Premier UPLC (Waters Co.) using a flow rate of 0.3 mL/min and an injection volume of 10 μL. All cIMS data were processed for general visualization and comparison using MassLynx v4.2 (Waters Co.). The space charge effect within the cIMS cell was normalized by adjusting the dynamic range enhancement lens to maintain a base peak intensity between 1 × 10^4^ and 5 × 10^4^ counts per second before each acquisition. Data were acquired for 1 min for all experiments unless stated otherwise.

### Optimization of the cIMS Conditions

All standards were dissolved in water/acetonitrile (95:5, *v/v*) at a concentration of 1 μg/mL. The ionization efficiency, fragmentation yield, and ion mobility performance were assessed for lactose using three of the most commonly used adduct forms for oligosaccharide analysis: sodiated and lithiated adducts in the positive mode and deprotonated adducts in the negative mode. For sodium and lithium adduction, the samples were spiked with 0.2 mM NaI or LiCl, respectively. Lactose reduced with sodium borohydride (lactitol) was used as control to exclude the coexistence of anomeric forms.^20^

cIMS settings were optimized for the separation of monosaccharides and disaccharides individually by varying the height of the traveling wave from 5 to 50 V and varying the traveling wave velocity between 100 and 2500 m/s. During the optimizations, the separation time was set to 1 ms to ensure the ion mobility path length was limited to a single pass with all settings. The lithiated anomers of glucose were used as a benchmark for monosaccharide anomer separation, for which the highest degree of separation was achieved at a wave height of 35 V and a wave velocity of 1500 m/s. These settings were used for all of the following monosaccharide separations. The separation of disaccharide isomers was similarly optimized using lithiated lactose and galactobiose, for which the best separation was achieved at a wave height of 50 V and a wave velocity of 1500 m/s. These conditions were used for further disaccharide separations.

### Deconvolution of Overlapping Disaccharide Anomers

The anomers of disaccharides could not be visually separated by any number of passes through the cIMS using the previously optimized conditions. To determine drift times for these anomers, CID fragmentation post-cIMS was performed for deconvolution. To process post-cIMS fragmentation data, the raw data were converted to mzXML format using MSConvert from the proteowizard suite.^100^ Further processing and deconvolution was performed in MATLAB 2019b (The MathWorks Inc., Natick, MA) using the Bioinformatics Toolbox and the MCR-ALS Toolbox.^101^ An altered form of the ROIpeaks algorithm from the MCR-ALS toolbox was used for the detection of regions of interest. For all MCR-ALS deconvolutions, forced to zero non-negativity constraints were used on the concentration profiles and spectra, and a horizontally implemented unimodality constraint was used on the concentration profiles with a tolerance of 1.1. All mass spectra were normalized to an equal height of 1. The MCR-ALS algorithm was set to a convergence criterion of 99%. Pure component mobilograms were reconstructed from the deconvolution results by multiplying the concentration profile vectors by the sum of their pure component spectrum vectors.

### Identification of Disaccharide Anomers Using cIMS^2^

Determination of the anomeric configuration of incompletely resolved disaccharide anomers was developed for galactobiose using tandem ion mobility spectrometry (cIMS^2^).^28^ Lithiated galactobiose (*m*/*z* 349.13) was isolated using the quadrupole. The reducing-end anomers were partially separated using the monosaccharide-optimized ion mobility method. A 0.5 ms slice of either the fronting or trailing end of the peak in the precursor ion mobilogram was transferred to the prearray store of the cIMS, and the rest of the ions were ejected from the cIMS cell. The stored ions were reinjected under collisional activation by a voltage gradient between the prearray store and the cIMS entrance, which was optimized in steps of 5 V for each slice to achieve the highest lithiated monosaccharide (*m*/*z* 187.08) fragment yield. All cIMS^2^ spectra were acquired over 5 min to increase spectral clarity. The resulting fragment ions were separated for the same time as the monosaccharide standards to compare the monosaccharide standard spectra to the cIMS^2^ fragment data.

## Results and Discussion

### Selection of the Ionization Mode

**Figure 1:**
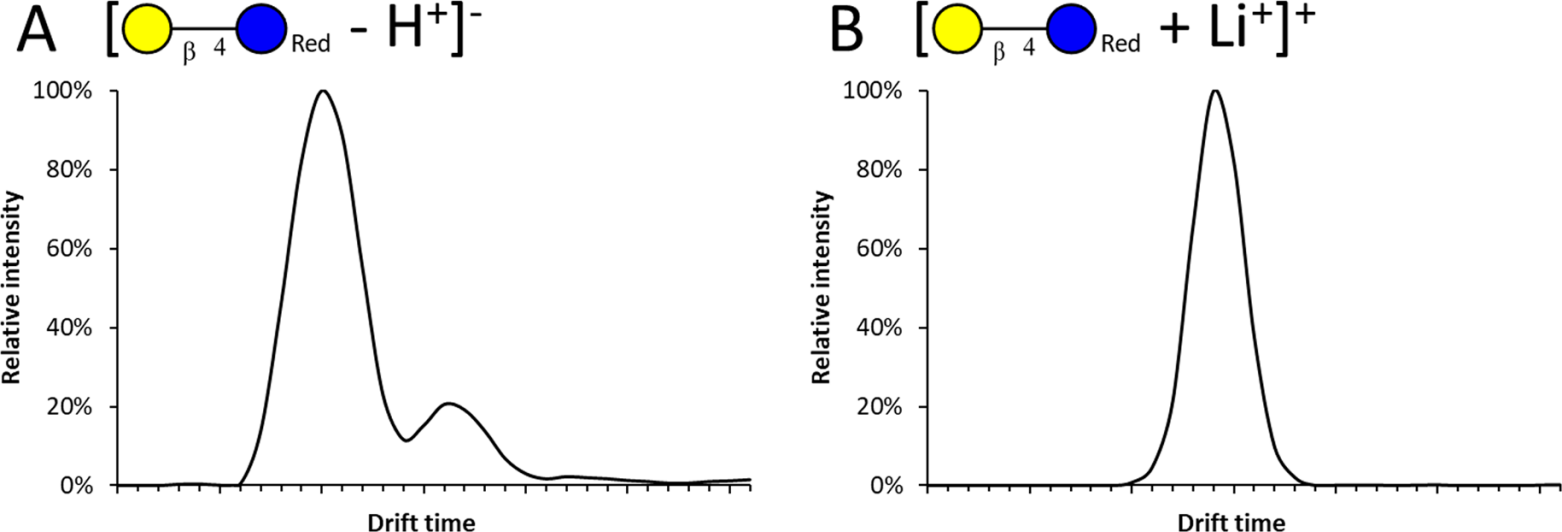
Ion mobilograms of (A) deprotonated reduced lactose in the negative mode and (B) lithiated reduced lactose in the positive mode.

The most common ionization modes for oligosaccharide mass spectrometry are deprotonation in the negative mode^29−31^ and sodium^32^ and lithium^33^ adduction in the positive mode. These modes were assessed for their suitability for cIMS and cIMS^2^ in terms of ionization efficiency, fragment yield, and ion mobility behavior. The ionization efficiency and fragment yield are favorable for deprotonation compared to metal ion adduction, since adduct desorption does not exist for deprotonated ions. However, multiple mobility peaks were found in the negative mode for deprotonated reduced lactose, which cannot constitute different isomeric forms (Figure 1A). Only one peak was observed in positive mode for adducted (lithiated) reduced lactose (Figure 1B).

Oligosaccharides contain multiple protons that can be lost upon deprotonation to result in multiple charge-site isomers (deprotomers) with distinct spatial structures, which have been reported to be separable by cIMS.^34^ This explains the multiple peaks observed in the negative mode. Other studies suggested that fast migration of the charge site occurred in deprotonated oligosaccharides, resulting in an averaged drift time and not in separable deprotomers.^31^ The present study indicates the existence of persistent separable deprotomers of (reduced) disaccharides complicating the identification of oligosaccharides, as multiple deprotomers of both precursor and fragment ions could be formed. Therefore, adduction in the positive mode, which yielded one distinct peak, suggesting the absence of (separable) charge-site isomers, was deemed favorable for further studies.

**Figure 2:**
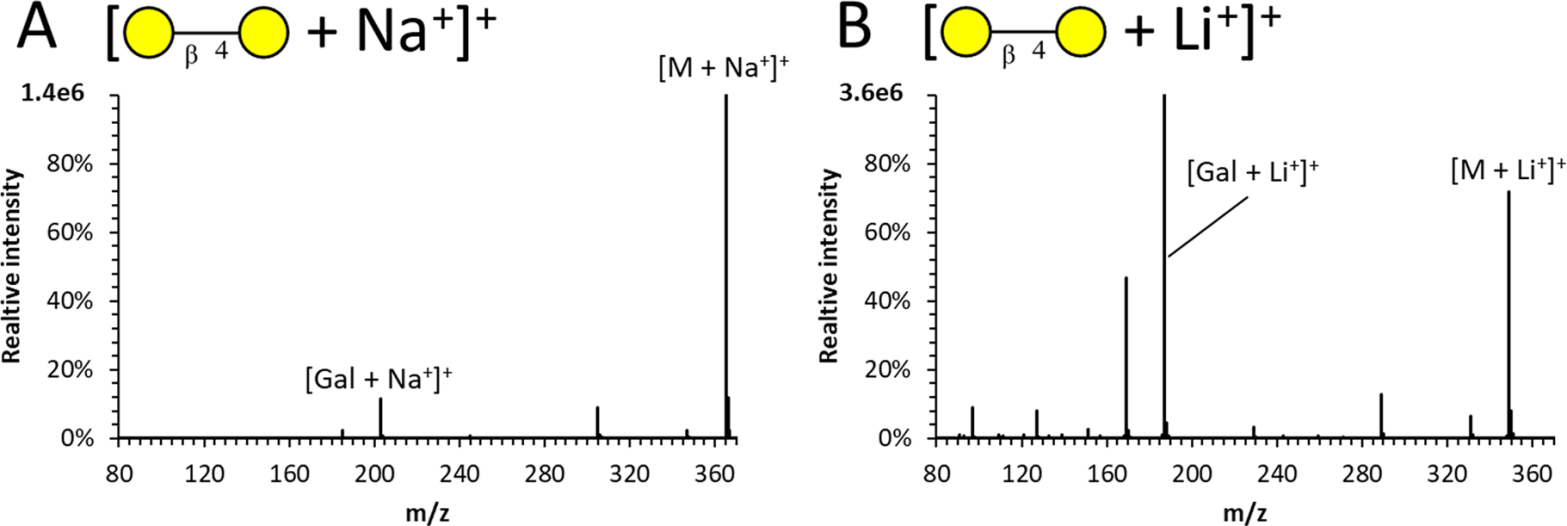
CID fragmentation spectra of (A) sodiated and (B) lithiated galactobiose acquired by using a transfer collision energy of 35 V.

The fragmentation behavior of sodium and lithium adduct ions of carbohydrates were compared using (β1–4) galactobiose. The ionization efficiencies for the sodiated and lithiated ions were found to be comparable. However, the glycosidic (monosaccharide) fragment yield from lithiated disaccharide ions was more than 20× higher (Figure 2). This is in line with previous studies.^35^ Lithium, being a smaller ion, is more strongly bound to the analyte, allowing fragmentation at increased collision energies without significant ion losses due to adduct desorption.

The higher fragment yield of the lithiated ions was deemed to be more important for the following fragmentation-driven characterization strategies. Therefore, further experiments were performed in positive mode on the lithiated ions.

### cIMS-MS Separation of Monosaccharides and Their Anomers

**Figure 3:**
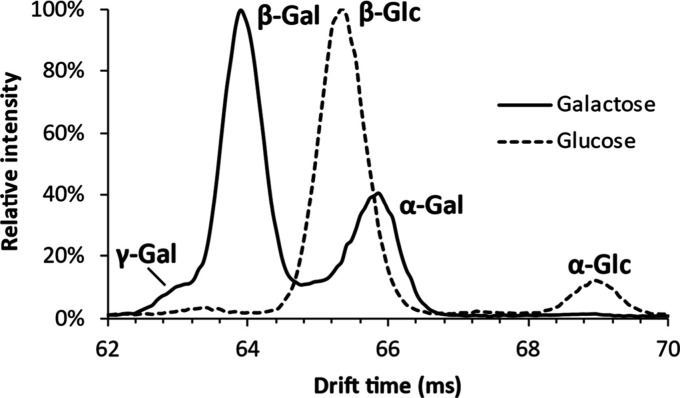
Overlayed ion mobilograms of the separated anomers of lithiated glucose and galactose.

The number of cIMS passes was optimized for the separation of galactose and glucose anomers by increasing the separation time until the isomers were clearly separated. cIMS settings were optimized for the α- and β-anomers of glucose and found at a wave height of 35 V and a wave velocity of 1500 m/s. Using these settings, baseline separation of glucose anomers was achieved (Figure 3) with a separation time of 51 ms (three passes).

The separated anomers were identified by their relative intensities by comparison with the anomeric ratios of glucose and galactose in solution as determined by Campbell and Bentley.^36^ For galactose, our anomeric ratios (31.9% α, 62.6% β, and 5.4% γ; with γ representing a mixture of furanose forms) (Figure S1A) agreed well with those found in Campbell and Bentley’s study (31.5% pyranose α, 63.7% pyranose β, and 4.7% γ). For glucose, a discrepancy existed between the anomeric ratios determined here (9.3% α, 90.7% β) (Figure S2) and those from the literature (39.8% α, 60.2% β). This could be explained by a difference in the solvent system (100% H_2_O versus 5% acetonitrile in H_2_O) as well as by the unpredictable effects of the heat, pressure, solvent composition, and charge density in the ESI spray droplets. It is, however, unlikely that these variables would differentially affect galactose and glucose to this extent. We did find that both the total intensity and the detected anomeric ratio of glucose depended strongly on the applied collision energies. The proportion of α-glucose peaked at 27.2% when using a cone potential of 50 V (Figure S3), which is much closer to the value reported in the literature. We hypothesize that some analyte ions remain clustered with other solutes following ionization, the propensity of which is seemingly higher for α-glucose than for β-glucose. Applying an increased cone potential improves the desorption of analytes from these clusters and rectifies the apparent disparity between the observed anomeric ratio of glucose using cIMS and those reported in literature. The detected anomeric ratio was not found to change much for galactose between cone potentials of 10 and 80 V (Figure S1B). However, using a cone potential of 80 V, an additional peak is visible between the α- and β-anomers. Using post-cIMS CID, this peak was found to fragment much more readily than the α- and β-anomers. We expect this component to be an open ring form of galactose, which was previously proposed to be an intermediate for cross ring fragmentation reactions.^37^

### CID-cIMS-MS to Determine the Monosaccharide Composition of Disaccharides

**Figure 4:**
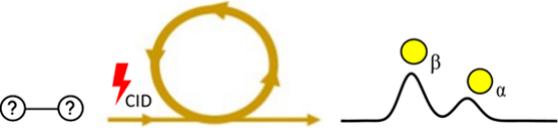
Graphical representation of the CID-cIMS-MS determination of the composition of galactobiose.

A graphical representation of the CID-cIMS approach used to determine the monosaccharide composition of disaccharides, as discussed in this section, is shown in Figure 4.

**Figure 5:**
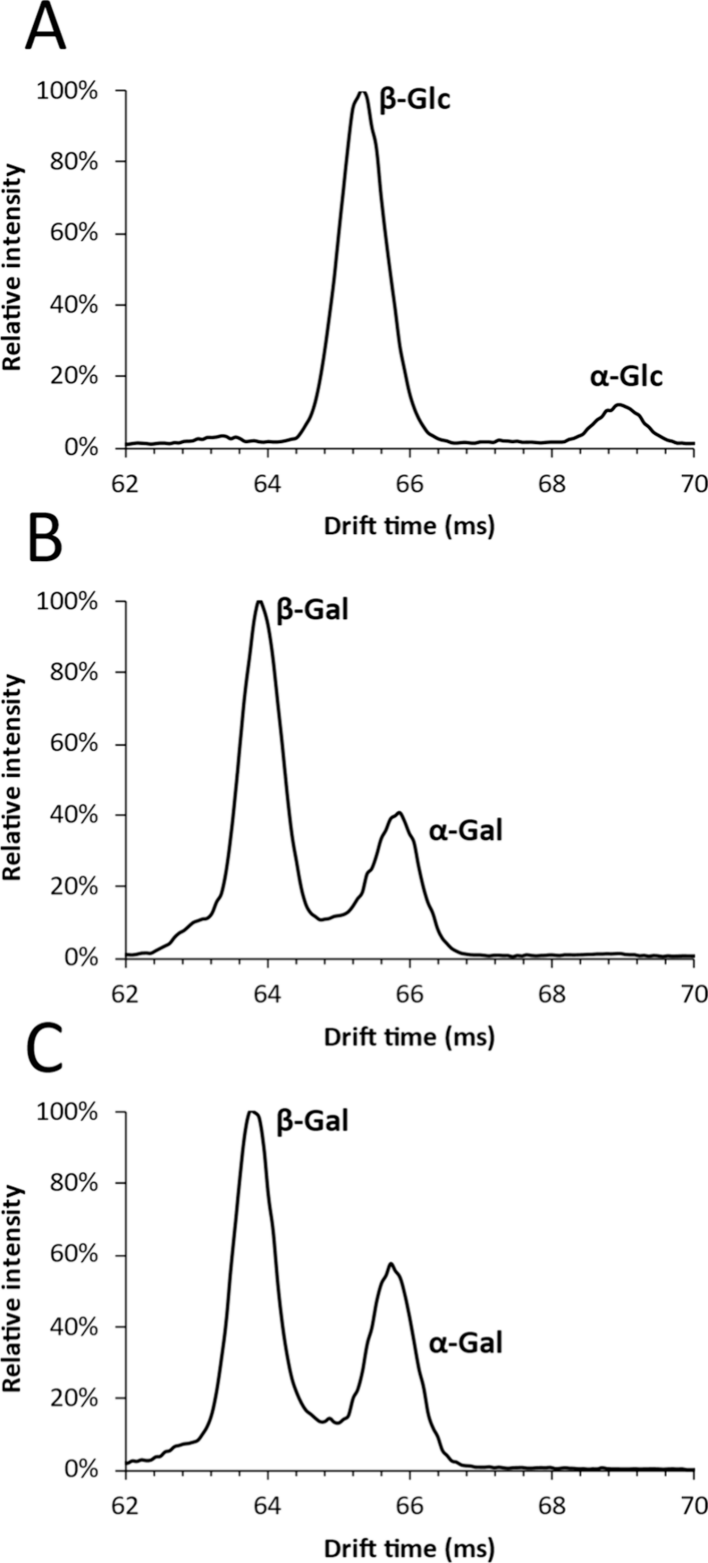
Comparison of ion mobilograms of (A) glucose and (B) galactose monosaccharide standards with (C) the monosaccharide fragments of galactobiose.

To demonstrate the composition determination of disaccharides, galactobiose (β-d-Galp-(1 → 4)-d-Galp) was infused and the lithiated precursor mass (*m*/*z* 349) was isolated using the quadrupole to exclude any interfering ions. The isolated disaccharide ions were fragmented in the trap collision cell before being transferred to the cIMS, and the resulting ions were separated using the monosaccharide-optimized method (Figure 5). The drift times of the monosaccharide fragments of galactobiose were identical to the drift times of the composing monosaccharide anomers. With these findings, we demonstrate that the disaccharide composition, including the anomeric configuration, can straightforwardly be determined by comparing drift times of monosaccharide fragments with drift times of monosaccharide standards. In contrast to the larger oligosaccharides studied used by Ollivier et al.,^27^ the complete composition and anomeric configuration of disaccharides can be determined by a single fragmentation step.

### Post-cIMS CID with Deconvolution Enables Recognition of Disaccharide Anomers

**Figure 6:**
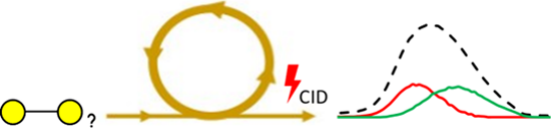
Graphical representation of post-cIMS-CID recognition of the anomers of galactobiose.

A graphical representation of the post-cIMS-CID approach with deconvolution that was used in this section to recognize disaccharide anomers is shown in Figure 6.

**Figure 7:**
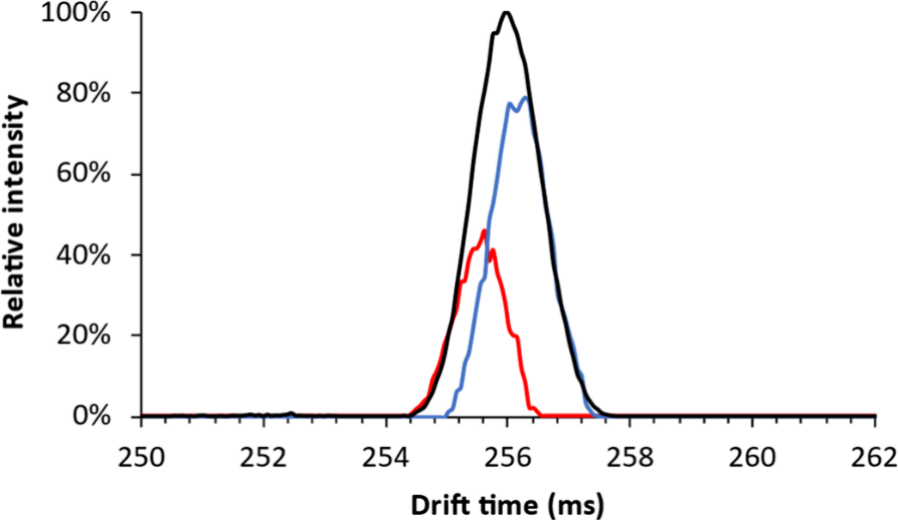
Ion mobilogram of the unresolved reducing end anomers of galactobiose (black); colored overlays represent the pure component mobilograms of the leading/faster (red) and trailing/slower (blue) anomers acquired using post-cIMS CID.

The described CID-cIMS-MS method could be applied for identification of the terminal monosaccharide units of trisaccharides or even larger oligosaccharides. In theory, using multiple fragmentation steps, internal compositions could also be probed. However, for complex samples where chromatographic separation is required, this would be unfeasible as the increasing scan time can cause undersampling of the chromatographic separation. Furthermore, all linkage type information would be lost when only monosaccharides can be used as reference compounds. On the contrary, a database of disaccharide mobilograms would help to characterize trisaccharides, as only a single fragmentation step is needed to obtain all structural information. To this end, the drift times of disaccharide reducing-end anomers need to be known. However, the anomeric ratios of most disaccharides are not available from the literature. Furthermore, we found that disaccharide anomers were significantly more difficult to resolve than monosaccharide anomers. No visible separation was achieved even after a separation of 230 ms under optimized conditions (Figure 7). Increasing the separation time further caused the spatial width of the peak to exceed the length of the cIMS cell, causing the head of the peak to overlap with its tail (also termed wrap-around), indicating the practical limit of the instrument. However, the absence of visible separation does not mean that no separation occurred.

The presence of partially separated anomers for galactobiose was shown by using fragment-based deconvolution by applying a CID potential of 30 V in the ion transfer cell after the cIMS fragmentation spectra for each point in the mobilogram were recorded. The data was processed using multivariate curve resolution alternating least-squares (MCR-ALS) using two components. The pure ion mobility profiles can be used to determine the drift times of the partially separated anomers.

### Identification of the Anomeric Configuration of Oligosaccharides Using cIMS^2^-MS

**Figure 8:**
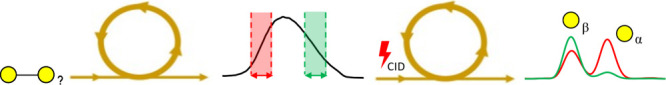
Graphical representation of the cIMS2-MS determination of the drift time order of the anomers of galactobiose.

A graphical representation of the IMS^2^ approach applied in this section to identify the anomeric configuration of oligosaccharides is shown in Figure 8.

In the previous section, we demonstrated how overlapping disaccharide anomers can be deconvoluted via post-cIMS CID. However, the anomers cannot be identified based on such data. To identify the anomers, cIMS^2^ was performed. A fraction of the ion mobilograms of the partially separated disaccharide anomers was collected in the prearray store before the ion mobility cell. Other ions were then ejected, and the stored ions were reinjected under CID conditions. The fragments formed were then separated by cIMS, resulting in cIMS^2^ analysis. Since the same wave height, wave velocity, and separation time settings are used, the cIMS^2^ mobilograms corresponding to the monosaccharide fragments are comparable to the previously recorded monosaccharide mobilograms.

**Figure 9:**
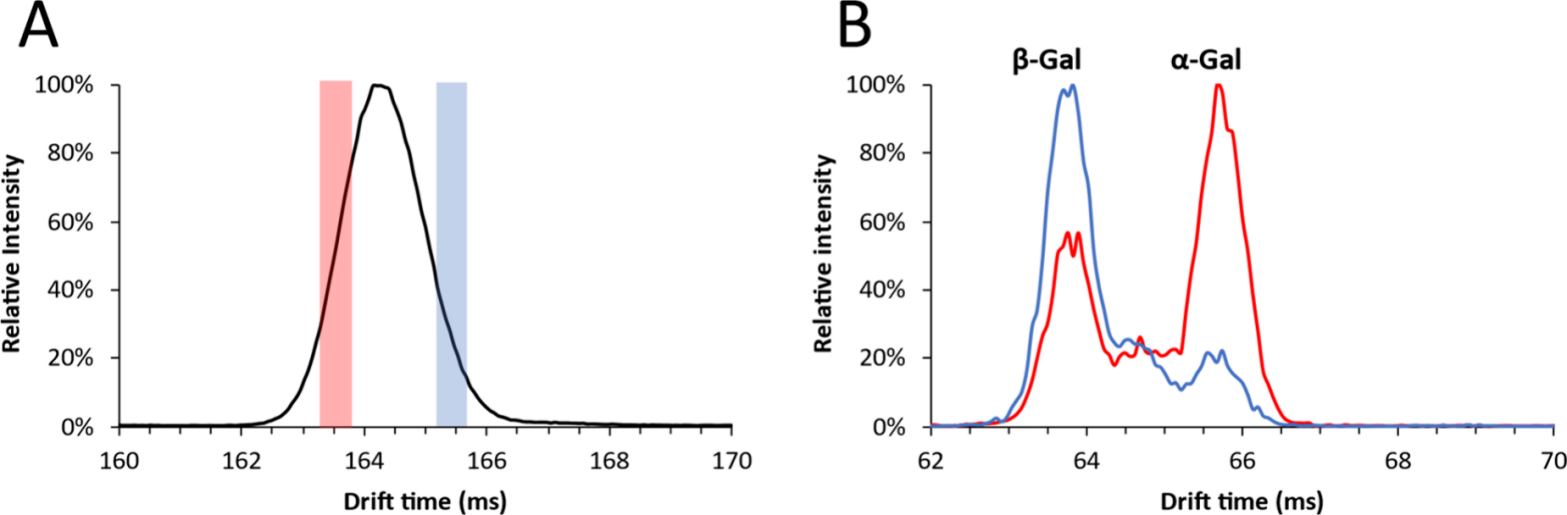
(A) Mobilograms of galactobiose acquired using the monosaccharide method and a separation time of 160 ms. Colored bars indicate the isolated regions for the leading (red) and trailing (blue) regions for cIMS^2^. (B) cIMS^2^ mobilogram of monosaccharide fragments (*m*/*z* 187.08) of galactobiose from the leading (red) and trailing (blue) isolation windows.

Figure 9 shows the identification of the anomeric configurations of galactobiose anomers. The red and blue overlays in the precursor mobilogram (Figure 9A) show the isolated regions corresponding to the respective red and blue monosaccharide fragment mobilograms (Figure 9B). Although there is overlap of the disaccharide anomers and the fragment mobilograms are not pure, clear distinctions can be made. The leading (higher mobility) galactobiose anomer formed a higher number of α-galactose fragments and thus corresponds to β-d-Galp-(1 → 4)-α-d-Galp, whereas the trailing (lower mobility) galactobiose anomer formed a higher number of β-galactose fragments and thus corresponds to β-d-Galp-(1 → 4)-β-d-Galp. The minor presence of α-galactose in the trailing peak is likely due to the partial inclusion of the leading peak in the cIMS^2^ isolation window. As was observed for galactose at elevated cone potentials (Figure S1), a peak between the α- and β-anomers of galactose was detected, which presumably constitutes an open-ring form of galactose.

### Lactose and Cellobiose Identification Using cIMS

The methodologies we developed using galactobiose were also applied to the glucose-containing disaccharides lactose and cellobiose. Hereby we aimed to identify and determine the drift times of the disaccharide reducing end anomers for the population of a database

**Figure 10:**
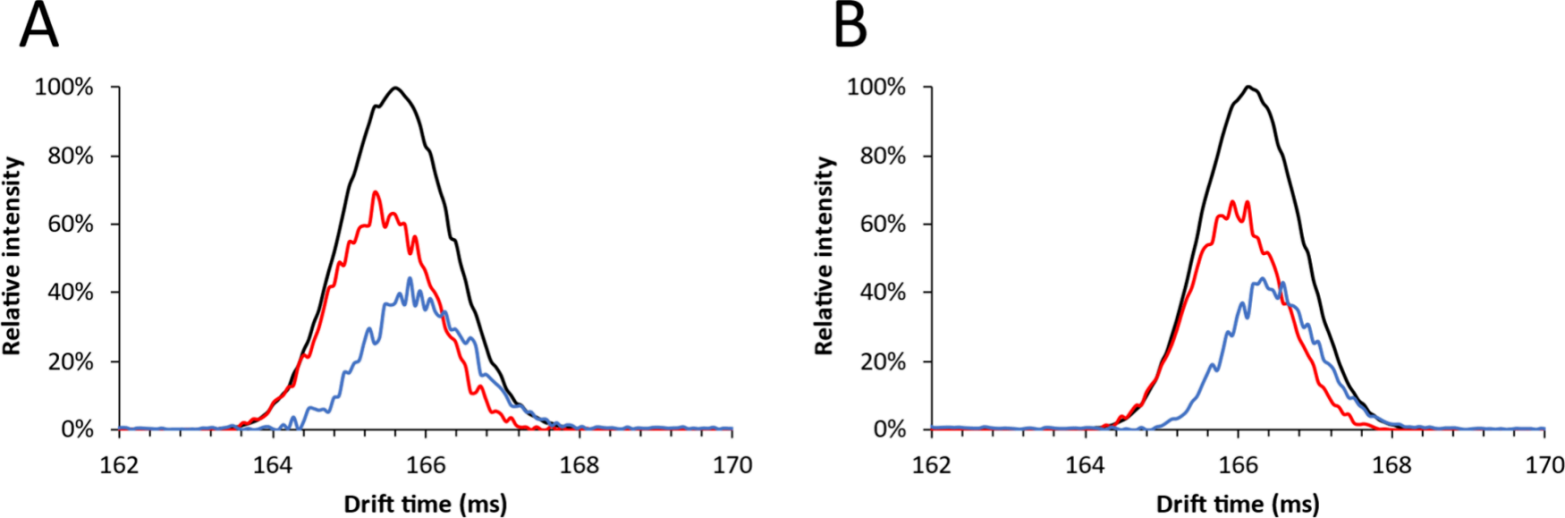
Mobilograms of (A) lactose and (B) cellobiose acquired using the disaccharide optimized method (black); colored overlays represent the pure component mobilograms of the leading/faster (red) and trailing/slower (blue) anomers acquired using post-cIMS CID.

The reducing end anomers of lactose and cellobiose were partially separated using the disaccharide optimized method and a separation time of 150 ms. Then a 30 V transfer CID potential was applied to induce post-IMS fragmentation. The obtained mobilograms were deconvoluted using the previously mentioned settings. The deconvolution results are presented in Figure 10.

**Figure 11:**
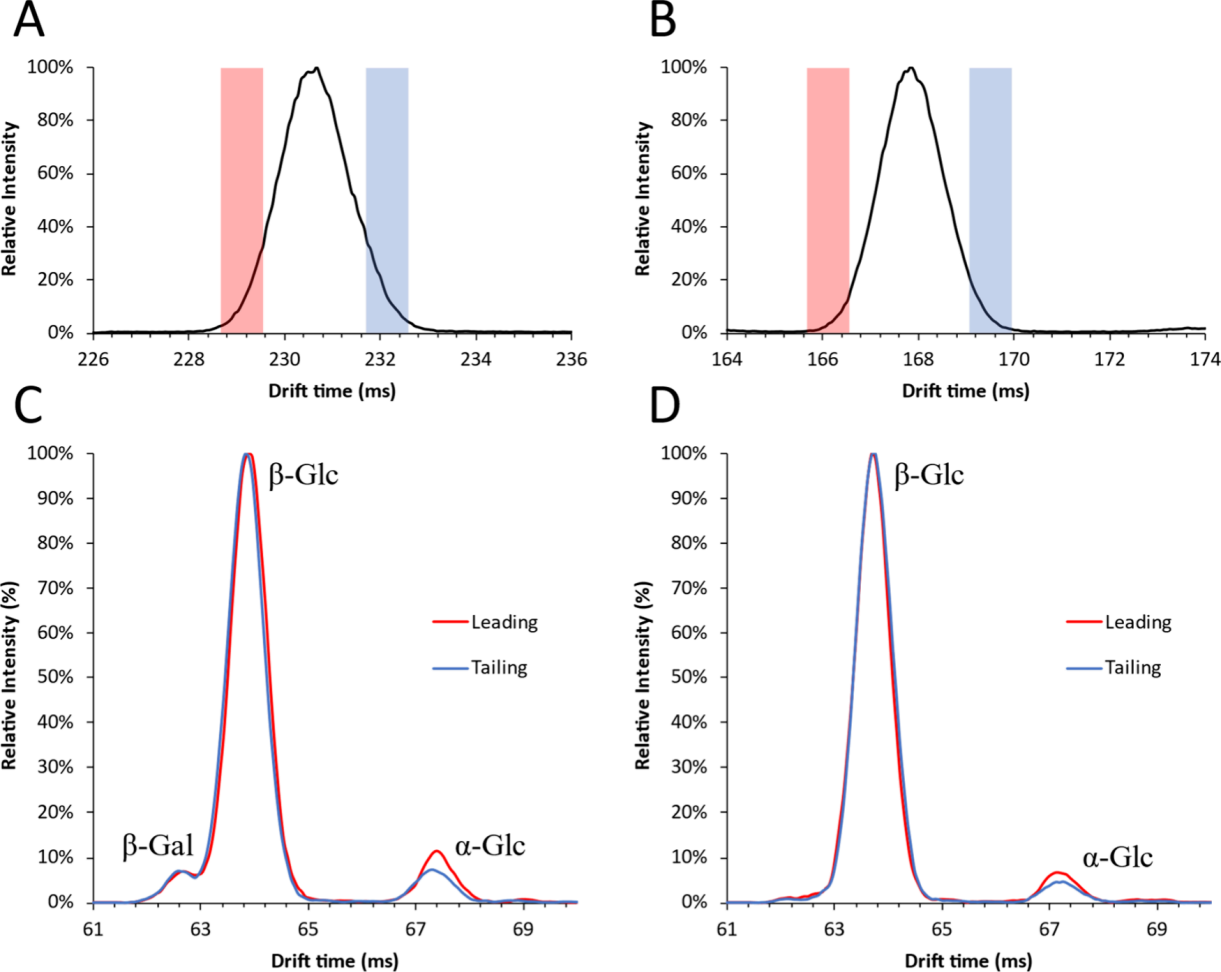
mobilograms of (A) lactose and (B) cellobiose acquired using the monosaccharide method and separation times of 225 and 160 ms, respectively. Colored bars indicate the isolated regions for the leading (red) and trailing (blue) regions for cIMS^2^. cIMS^2^ mobilograms of monosaccharide fragments (*m*/*z* 187.08) of (C) lactose and (D) cellobiose from leading (red) and trailing (blue) isolation windows.

Lactose and cellobiose were also subjected to cIMS^2^ to identify the partially separated anomers as described previously, the results of which are displayed in Figure 11. Compared to galactobiose (Figure 9), the monosaccharide cIMS^2^ mobilograms of lactose and cellobiose showed little difference between the leading and trailing ends of the cIMS^1^ peak. However, even with these small differences, it can still be concluded that for both lactose and cellobiose the leading (faster) component constitutes the α-anomer due to the higher relative contribution of α-glucose in the cIMS^2^ mobilogram.

The trailing (slower) components constitute β-anomers. For all three of the tested disaccharides, the leading component constituted the α-anomer. In contrast to galactobiose, the pure component mobilograms resulting from lactose and cellobiose present a higher contribution of the α-anomer. This could indicate a higher concentration of the α-anomer in solution but could also be due to differences in the ionization efficiency or fragment yields of these anomers.

The accuracy of the drift times resulting from the deconvolution was validated by comparison of the fragmentation spectra resulting from the deconvolution with the spectra obtained from the extremities of the unresolved peak in the raw data, which represent the pure anomers (Figure S4). The MCR-ALS algorithm minimizes the difference between the raw data matrix and the product of the pure component spectra and concentration profiles. The accurate representation of the pure component spectra indicates correct deconvolution. Drift time reproducibility is still a hurdle for multipass cIMS separations, as calibration is hindered due to the absence of accurate reference CCS values.^28^ Since the measurements of lactose and cellobiose were performed on a different day than galactobiose, the monosaccharide references for lactose and cellobiose were remeasured on the same day (Figure S5), allowing unambiguous annotation of the monosaccharide anomers. The drift time difference between cellobiose and lactose anomers was found to be in a range similar to that of the drift time difference found for galactobiose anomers. A first separation of the individual dimers was recognized and indicates the feasibility of deconvoluting mixtures of lactose and cellobiose anomers.

The successful detection and identification of disaccharide reducing end anomers demonstrated here will allow the construction of disaccharide databases for the subsequent identification of unknown trisaccharides.

## Conclusions

In this work, we have developed several complementary cIMS-MS approaches for the determination of the monosaccharide composition and the anomeric configuration of hard to separate GOS disaccharide isomers. This study, to our knowledge, presents the first example of the determination of the composition and anomeric configuration of disaccharides by their monosaccharide fragments using ion mobility spectrometry. Although the GOS disaccharides used in this study only consist of galactose and glucose, we are confident that our methods can also be applied to other disaccharides such as HMOs consisting also of *N*-acetylglucosamine and *N*-acetylgalactosamine. As such, our work lays the foundation to probe monomer identities and anomeric configurations within unknown oligosaccharides. The capability to assign disaccharide anomers in complex mixtures is vital in establishing true *de novo* identification using IMS-MS. This would be essential for IMS-based oligosaccharide identification, as disaccharides are the smallest possible structures that can describe all structural variations (composition, sequence, anomeric configuration, and linkage type) while still being feasible to acquire or synthesize to create a representative database. In addition, disaccharide fragments of larger oligosaccharides with a known structure could be used to further populate such a database. The utility of databases will ultimately hinge on the development of more reproducible calibration procedures for multipass cIMS methods.

The ability to probe the identity of monosaccharide fragments of an oligosaccharide at any stage of the characterization workflow will be tremendously useful for future sequencing studies. Moreover, the ability to distinguish and identify disaccharide isomers even when they are not well resolved by IMS will allow the population of disaccharide cIMS databases as well as the recognition of mixed spectra of disaccharide fragments.

## Supporting Information Available

Integrated ion mobilogram of galactose with cone potentials of 10 and 80 V; contribution of the α-anomer of glucose as a function of the cone potential; comparison of the fragmentation spectra from the leading and trailing extremities of the peak in the raw data and the pure fragmentation spectra obtained through deconvolution for the anomers of galactobiose, lactose, and cellobiose; and monosaccharide reference mobilograms of galactose and glucose for IMS2 identification of lactose and cellobiose (PDF)
