# Supplementary material for: Structural Characterization of Disaccharides Using Cyclic Ion Mobility Spectrometry and Monosaccharide Standards
Source: J Am Soc Mass Spectrom. 2024 Apr 18;35(5):1012–20. doi: 10.1021/jasms.4c00029 (PMC11066964; doi:10.1021/jasms.4c00029)
Supplement: Supplementary file 1 — js4c00029_si_001.pdf [file js4c00029_si_001.pdf]

Structural characterization of disaccharides using cyclic ion mobility spectrometry and monosaccharide standards

Bram van de Put, Wouter J.C. de Bruijn, Henk A. Schols*

Laboratory of Food Chemistry, Wageningen University, Bornse Weilanden 9, 6708, WG, Wageningen, the Netherlands

* corresponding author (Wouter.debruijn@wur.nl)

Supplementary data

Table of contents

Figure S1 Integrated ion mobilogram of galactose with cone potentials of 10 V (A), and 80 V (B). The peak marked with an asterisk is suspected to be an open ring configuration created by collisional excitation.

Figure S2 Integrated ion mobilogram of glucose with respectively the: beta and alpha forms.

Figure S3 The contribution of the alpha anomer of glucose as a function of the cone potential. The dotted line indicates the value reported in literature (36%).

Figure S4 Comparison of the fragmentation spectra of the the leading (red) and trailing (blue) extremities of the peak in the raw data, and the pure fragmentation spectra obtained through deconvolution for the anomers of galactobiose, lactose and cellobiose.

Figure S5 Monosaccharide reference mobilograms of galactose and glucose for IMS2 identification of lactose and cellobiose.

21

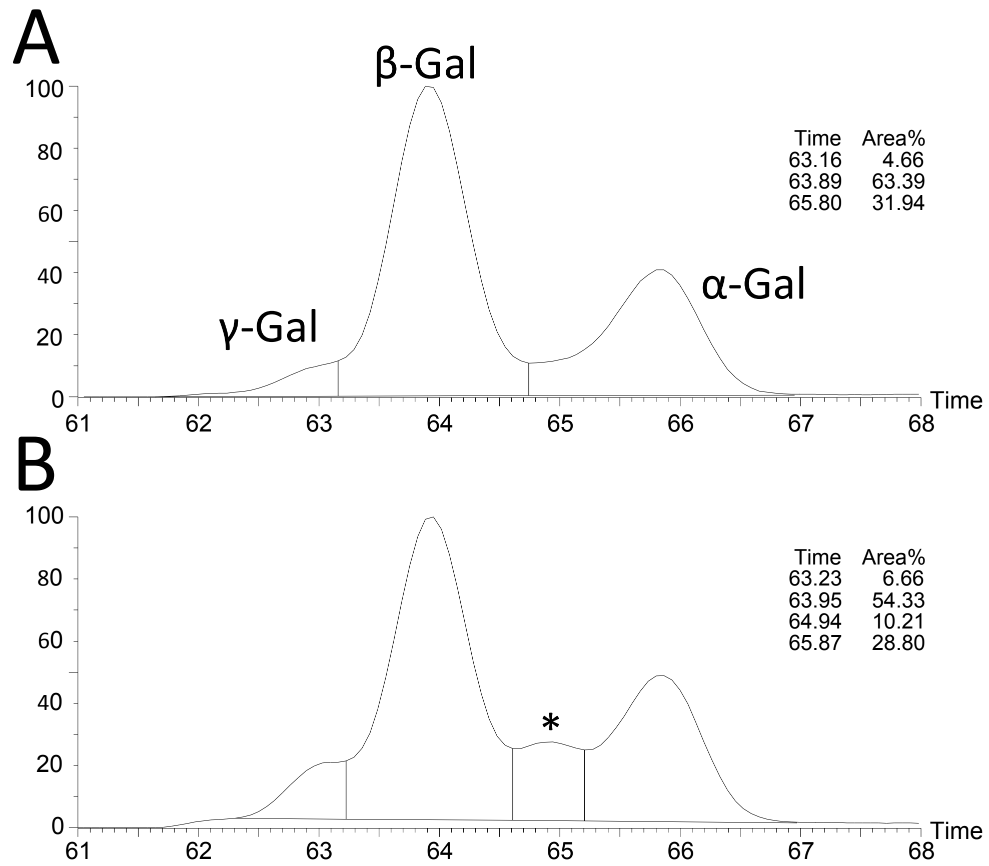

Figure S 1: Integrated cIMS mobilogram of galactose with cone potentials of 10 V (A), and 80 V (B). The peak marked with an asterisk is suspected to be an open ring configuration created by collisional excitation.

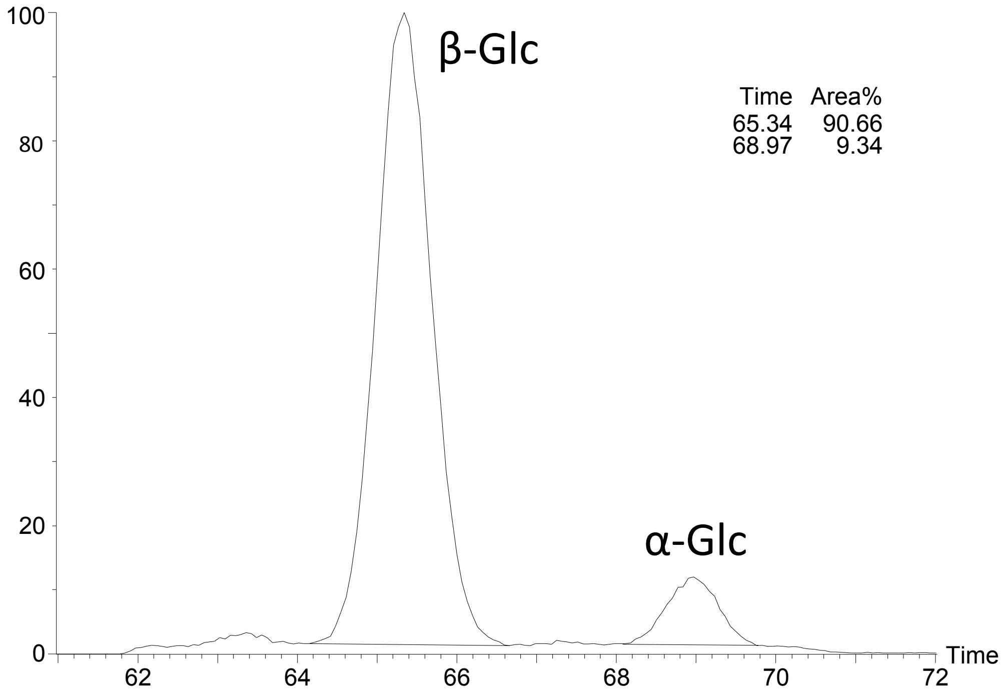

Figure S 2: Integrated ion cIMS mobilogram of glucose with respectively the: β and α forms.

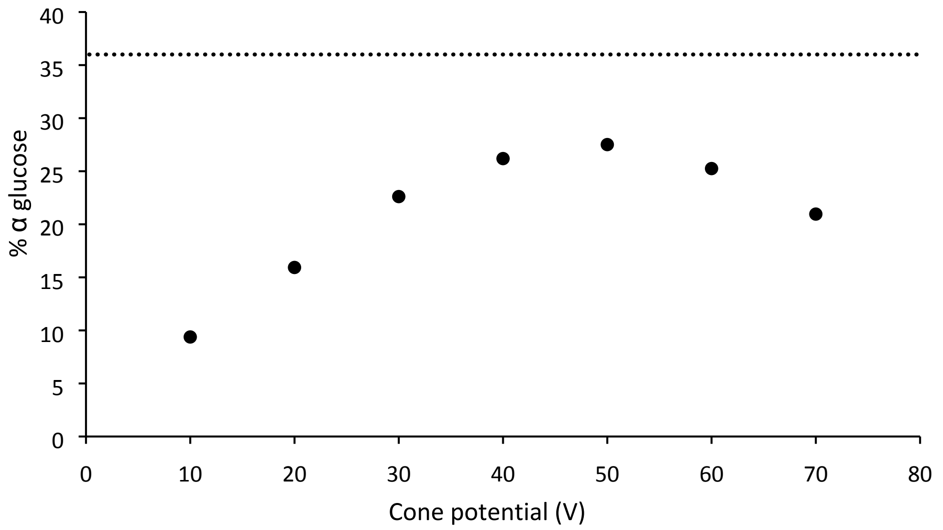

Figure S 3: The contribution of the α anomer of glucose as a function of the cone potential. The dotted line indicates the value reported in literature (36%).

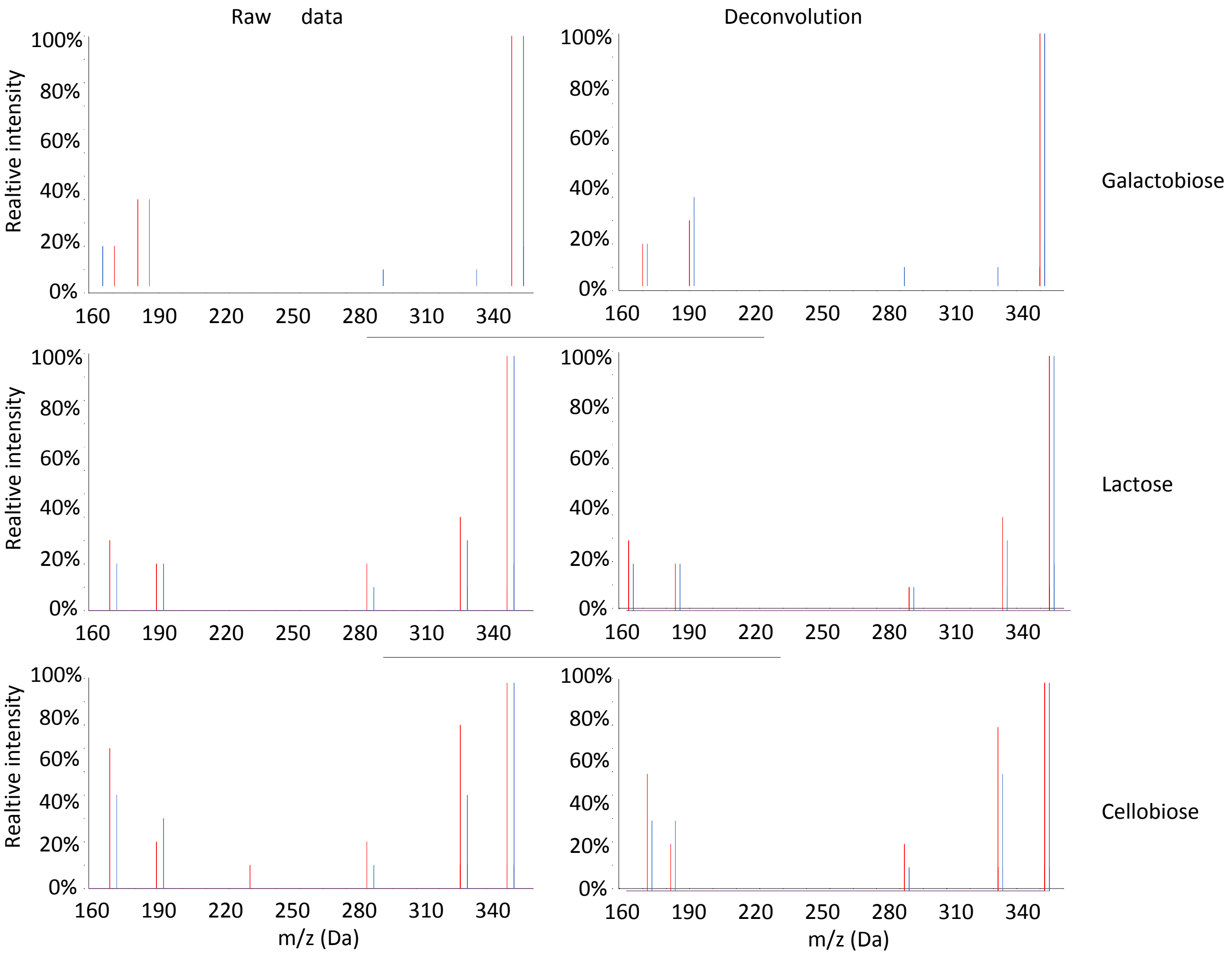

Figure S 4: Comparison of the fragmentation spectra of the the leading (red) and trailing (blue) extremities of the peak in the raw data, and the pure fragmentation spectra obtained through deconvolution for the anomers of galactobiose, lactose and cellobiose.

24

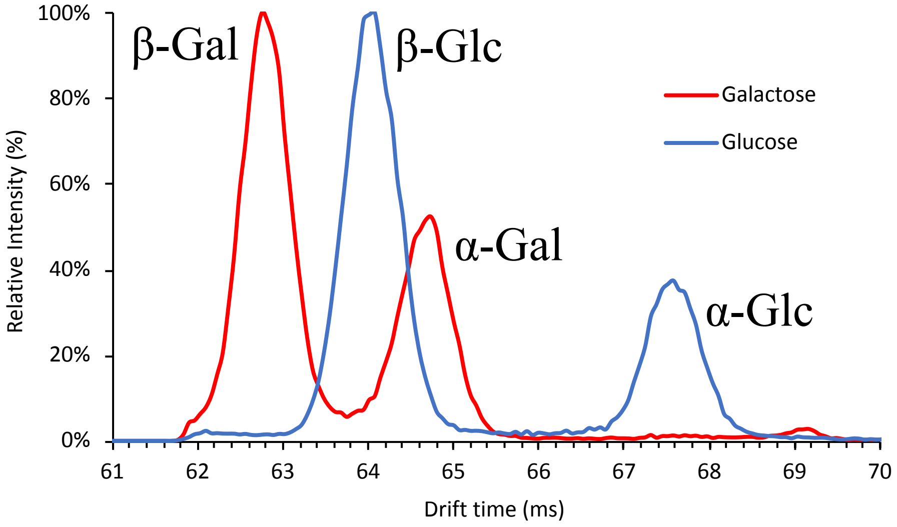

Figure S 5: Monosaccharide reference mobilograms of galactose and glucose for IMS2 identification of lactose and cellobiose.
